# 6-Amino Caproic Acid-Modified CuFe_2_O_4_ Nanocomposite for Amaranth Dye Removal: Optimization, Thermodynamics, and Isotherm Studies

**DOI:** 10.3390/nano16040228

**Published:** 2026-02-10

**Authors:** Rabia Ahmed, Ghaida H. Munshi, Abeer Mohammed Al-Balawi, Salwa D. Al-Malwi, Azza A. Al-Ghamdi, Reema H. Aldahiri, Rita Rajput, Bushra Fatima, Elham A. Alzahrani, Sumbul Hafeez

**Affiliations:** 1 Department of Chemistry, Jamia Millia Islam, New Delhi 110025, India; rahmad@jmi.ac.in (R.A.); rajputrita93@gmail.com (R.R.); bushrafatima89@gmail.com (B.F.); 2 Department of Chemistry, College of Science, University of Jeddah, Jeddah 21959, Saudi Arabia; gmunshi@uj.edu.sa (G.H.M.); aaalgamdi@uj.edu.sa (A.A.A.-G.); rhal-dhahery@uj.edu.sa (R.H.A.); 3 Department of Chemistry, University Duba College, University of Tabuk, Tabuk 71491, Saudi Arabia; ab_albalawi@ut.edu.sa; 4 Department of Chemistry, College of Science, Northern Border University, Arar 91431, Saudi Arabia; salwa.almaalawi@nbu.edu.sa; 5 Department of Chemistry, College of Science, University of Ha’il, Ha’il 81451, Saudi Arabia; 6 Department of Civil and Environmental Engineering, Villanova University, Villanova, PA 19085, USA

**Keywords:** copper ferrite, amino acid, caproic acid, nanocomposite, adsorption, amaranth

## Abstract

In this work, 6-aminocaproic acid-modified copper ferrite nanoparticles were synthesized and used as an adsorbent for removing Amaranth dye from water. The modified nanoparticles were easily prepared using a simple and cost-effective method, namely the coprecipitation method. The nanocomposite was characterized by techniques like FTIR, XRD, SEM with EDS, and TEM. To evaluate the adsorption capacity of Amaranth dye, concentrations of Amaranth, contact time, nanocomposite dose, and solution pH were optimized. Further, Langmuir and the Freundlich isotherm models have been investigated. Among these, the Freundlich model showed the most accurate correlation with the experimental result, indicating a multilayer and physico-chemical adsorption of Amaranth dye onto the heterogeneous surface of the prepared nanocomposite. The Langmuir maximum adsorption capacity of this study was ~17 mg g^−1^. Thermodynamic parameters (∆G° and ∆H°) confirmed that the adsorption process was spontaneous and exothermic. Adsorption kinetic studies showed pseudo second-order fitting with the multi-step adsorption process. The current adsorption performance was best for the first two adsorption–desorption cycles.

## 1. Introduction

The increasing amount of dye in water is becoming a serious environmental problem, leading to continuous water pollution [1]. Contaminated water not only harms aquatic life and plants, but is also very harmful to the human body [2,3]. The main sources of dye-containing wastewater are industries such as the textile [4] and paper industries [5]. Industrial activity has increased with population growth, leading to a significant increase in dye production and use [5,6]. Dyes are primarily organic complex compounds containing the azo functional group [7]. Many dyes have been discontinued due to their toxicity [8]. The toxicity in dyes is primarily due to their benzadiene metabolites [9]. Dyes also exhibit mutagenic and carcinogenic effects [9]. Amaranth dye is one such dye that is highly toxic and has been shown to have significant effects on the human body [10]. Amaranth dye is an anionic red color azo dye, used as a food additive and to color cosmetics, paper, fibers, and leathers [11].

Various physical, chemical, and biological methods have been used to remove dyes from water, with adsorption being considered a preferred technique [12,13,14,15]. Adsorption is an inexpensive and easy-to-use technique that relies on surface phenomena. In the adsorption process, the adsorbate, or pollutant, is absorbed onto the surface of a solid (adsorbent) through functional groups or pores present on the surface [16,17]. This means that adsorption can be largely controlled by altering the solid surface and its functional groups [16,17,18]. Various materials have been used for dye adsorption, among which activated carbon has proved to be a widely used adsorbent [18,19]. However, the preparation of activated carbon requires high temperature and chemical action, which makes activated carbon overall expensive and also liable to secondary pollutants [18,19]. Alternative nanomaterials (NMs) to activated carbon have been discovered, as NMs that exhibit high surface area and are porous have proven to be better materials for adsorption [20,21]. Many NMs have been used so far, the most common being ferrite nanoparticles (NPs) of metal oxides (MOs) [22].

Many previous studies have shown that functional groups on the surface of NPs also help a lot in governing the adsorption [23,24]; hence, MONPs were modified in various ways to address the functional groups on their surface, which demonstrated better adsorption capacity [23,24,25]. Modifying MONPs surfaces with biodegradable acids is a hot topic at present, and many studies are underway to modify MO surfaces with biodegradable acids that create functional groups on the MO surface, which aid in adsorption [24,25]. Biodegradable materials are non-toxic and do not produce secondary pollutants in water [24,25,26].

In the past, NPs have been modified using biodegradable acids like phthalic acid [24] and citric acid [25], which demonstrated significant adsorption capacity. Biodegradable acids are naturally occurring organic substances that are converted by microorganisms into non-toxic by-products; therefore, they have no environmental impact [24,25,26].

The current study produced modified copper ferrite (CF), a previously reported adsorbent that has helped remove a variety of pollutants [27,28]. The synthetic biodegradable acid, 6-aminocaproic acid (6-ACA) was used to modify CF in this study. This acid has a carboxylic acid at one terminal and an amine group at the other, which easily modifies the NPs surface [29].

CF is chemically stable over a wide pH range and is easier to prepare than other NPs, making it a low-cost NP. Its large surface area also makes it an excellent choice for adsorption applications. Modifying CF with acids (6-ACA) can help overcome several drawbacks, such as increasing the dispersion of NPs in water, expanding their functional sites, providing further stability to NPs, and increasing their surface area while reducing aggregation. It can also enhance the biocompatibility of CF by reducing toxicity.

Therefore, in the present study, CF was modified with 6-ACA, and this product was used as a nano-adsorbent. In this study, Amaranth dye was removed, thermodynamics and isotherms were studied, and adsorption efficiency was optimized at various conditions.

## 2. Experimental

### 2.1. Materials and Methods

A.R. grade copper acetate monohydrate, NaOH, was purchased from Merck India (New Delhi). Ferric chloride and 6-Amino Caproic acid were obtained from LOBA, India. Amaranth dye (Mol. Wt. = 604.743 g mol^−1^, λ_max_ = 520 nm) was procured from Sigma Aldrich Ltd., India. A stock solution of 100 mg L^−1^ of Amaranth dye was prepared and stored. The solution pH was changed by adding 0.1M hydrochloric acid and/or 0.1M sodium hydroxide using a digital pH meter (Eutech™ pHTestr30, ThermoFisher Scientific). Throughout the experiment work deionized water (pH = 7) was used. The UV/VIS (U-3900 spectrophotometer, India) spectrophotometer had been utilized to calculate Amaranth dye concentrations.

### 2.2. Synthesis of 6-ACA-CF

To prepare the 6-ACA-CF nanocomposite (NC), 100 mL of 0.1M ferric chloride solution was added to 100 mL of 0.05M copper acetate monohydrate solution under continuous stirring. The solution mixture was further stirred for 20 min on a hot plate magnetic stirrer at 50–55 °C. After 20 min of continuous stirring, to this solution mixture, 100 mL of 0.05 M 6-Amino Caproic acid solution was added, and the obtained solution was further stirred for 40 min on a hot plate magnetic stirrer at 50–55 °C. Afterwards, to this solution mixture, 8 M of NaOH solution was added drop by drop until the pH of the solution mixture reached 10. At this pH, a black color precipitate was obtained that was centrifuged at 3000 rpm using a centrifugal machine (REMI-8). The centrifuged sample was washed several times using deionized water, dried at 80 °C for 24 h, and crushed into a powdered form using a mortar pestle. The obtained sample was further investigated for physico-chemical properties and adsorption application.

### 2.3. Instrumentations and Characterization

The crystal structure of the obtained sample was characterized by using X-ray diffraction (XRD), performed on a PANalytical X’Pert diffractometer with Cu-Kα radiation (λ = 0.154 nm). The instrument is equipped with a Cu filter and a proportional counter detector, scanning over a 2θ range of 10° to 80° at room temperature and at 40 kV and 30 mA. The morphology, structural features, and particle size were further examined using transmission electron microscopy (TEM) with a Tecnai T-30 FEG-TEM, operating at 300 kV (imaged at 80 kV). Surface morphology and elemental composition were demonstrated by field emission scanning electron microscopy (FESEM, Nova Nano SEM 450) coupled with energy-dispersive X-ray spectroscopy (EDS, Bruker, 127 eV), operated at 5 kV. Additionally, Fourier transform infrared spectroscopy (FTIR) was conducted using a PerkinElmer Frontier FTIR spectrometer in the spectral range of 400–4000 cm^−1^ to identify functional groups and vibrational modes present in the material.

### 2.4. Determination of Point of Zero Charge, pH_pzc_

The pH_pzc_ of the current sample was measured at 28 °C by taking 0.20 g of sample in 20 mL of 0.1N KNO_3_ electrolyte solution. The initial pH of the solution was adjusted using 0.1 mol L^−1^ HCl and/or NaOH, followed by continuous stirring at 155 rpm for 5 h. After this period, the final pH was recorded. The pH_pzc_ of current sample was identified by plotting the difference between the initial and final pH values (ΔpHs = pH_initial–pH_final) against the initial pHs. Theoretically it is assumed that when the solution pH is below the point of zero charge (pH < pH_pzc_), the sample surface becomes positively charged, whereas at pH values above pH_pzc_ (pH > pH_pzc_), it acquires a negative charge. These changes in sample surface charges may influence the adsorptive interaction between the sample and the anionic Amaranth dye during the adsorption process. This influence may lead to the enhanced adsorption performance of the current sample under attractive conditions (opposite charges), and reduced performance, when repulsion occurs (like charges) [24].

### 2.5. Determination of Adsorption Capacity and Removal Rate

In this study, a batch process was used to investigate the adsorption of amaranth onto the 6-ACA-CF surface. A series of 50 mL Erlenmeyer flasks were taken, each containing 10 mL of an amaranth dye solution with a concentration of 10 mg L^−1^, and varying amounts (1.0–5.0 g L^−1^) of the 6-ACA-CF adsorbent were added to each flask. The pH of all solutions was maintained at 7.0. All the flasks were then shaken in a water bath shaker at 30 °C and 150 rpm for 120 min. In this experiment, the optimum dose was determined for a 10 mL solution with a concentration of 10 mg/L. Similarly, other variables such as temperature, time, pH, and concentration were also optimized, for which one parameter was varied while the other parameters were kept fixed.

After each set of experiments, the mixture of Amaranth dye solution and 6-ACA-CF in the Erlenmeyer flask was separated using centrifuge machine, and the supernatants were collected. Later, the supernatants were examined for the spectrophotometric analysis using UV–visible spectrophotometer at the absorption maximum of 580 nm. Through this analysis, the final concentration of Amaranth dye in the supernatant was determined and denoted as *C_e_*.

The equilibrium adsorption capacity (*Q_e_*) of Amaranth dye onto the 6-ACA-CF surface was calculated based on the dye concentration at equilibrium (final concentration) for each initial concentration, using the equilibrium adsorption capacity equation (Equation (1)) [25]:

(1) $$Q_{e}=\left(C_{o}-C_{e}\right)\frac{V}{m}$$

Here *C_o_* and *C_e_* represent the initial and equilibrium concentrations of Amaranth dye, respectively, *V* is the volume of the sample solution (in liters), and *m* is the mass of the adsorbent (in grams).

The percentage removal of Amaranth dye under influence of parameters such as doses of 6-ACA-CF, initial concentrations of Amaranth dye solution, solution pHs, reaction temperatures, and contact times were investigated using Equation (2) [30]:

(2) $$Removal\;\left(\%\right)=\frac{\left(C_{o}-C_{e}\right)}{C_{o}}100$$

The data obtained from these experiments were applied to thermodynamic, isotherm, and kinetic models, and the adsorption mechanism was explained in detail.

## 3. Results and Discussions

### 3.1. Characterization of 6-ACA-CF

**Figure 1:**
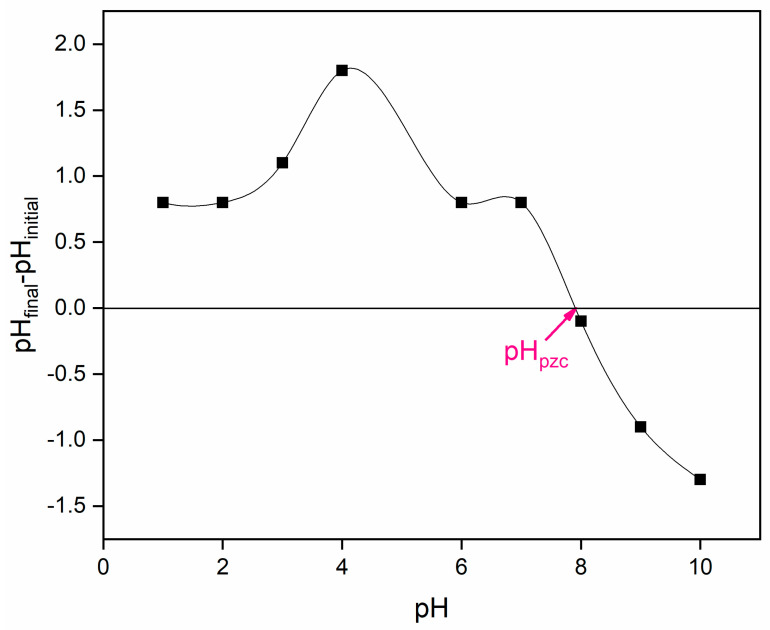
Figure of pH_pzc_ of 6-ACA-CF NPs.

The pH_pzc_ of 6-ACA-CF was found to be around ~7.9 (Figure 1). This pH_pzc_ refers to the pH at which a 6-ACA-CF surface immersed in an electrolyte solution carries no net electrical charge. This condition arises due to the balance between protonation and deprotonation processes occurring at the surface. At low pH levels (pH < pH_pzc_), protonation dominates, where the surface accepts H^+^ ions, resulting in a positively charged surface. In contrast, deprotonation involves dissociation of H^+^, giving a negative charge to a surface at higher pHs (pH > pH_pzc_) [31].

**Figure 2:**
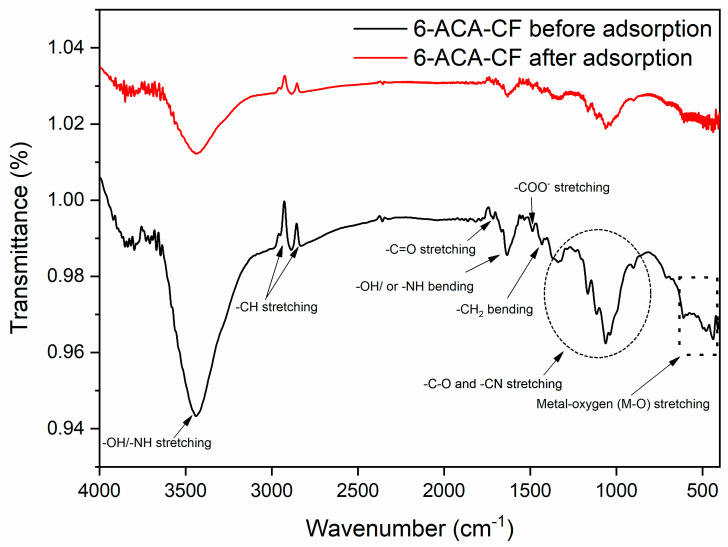
FTIR spectrum of 6-ACA-CF before adsorption (black line) and after adsorption (red line).

The FTIR spectrum of the prepared sample (Figure 2; black line) showed several functional groups as well as metal oxide peaks in the range of 4000 to 400 cm^−1^. The major absorption bands found in this FTIR spectrum included a broad absorption band between 3300 and 3500 cm^−1^, which corresponds to the overlapped frequency of N–H stretching vibration of primary amines and the O–H stretching vibration of carboxylic acids [31,32]. The broadness of the peak indicates strong hydrogen bonding. The peaks found in the region of 2930–2850 cm^−1^ is attributed to asymmetrical and symmetrical C-H stretching vibrations, primarily indicating the presence of aliphatic CH_2_ groups [30], which confirms the hexyl chain of 6-ACA. The stretching vibrations found around 1730 cm^−1^ indicate the C=O group of carboxylic acid, confirming the functionality of the COOH group [31,33]. Similarly, the peaks between the region 1630–1430 cm^−1^ showed N–H [33] bending vibrations and/or H–O–H bending vibrations (due to moisture) [34], COO^−^ vibrations [34], and CH_2_ bending vibrations [35]. The region from 1350 to 1000 cm^−1^ shows multiple peaks, indicating C–O stretching vibrations [33] and C–N vibrations [33]. The peak found near 700 cm^−1^ showed the rocking mode of CH_2_ [36]. All these peaks confirmed the functionalization of the sample by 6-ACA. In addition, some other peaks were found between 600 and 400 [37,38], which are basically metal–oxygen peaks, confirming the presence of copper ferrite.

The spectrum of the sample after adsorption of Amaranth dye was also analyzed (Figure 2; red line), and it was found that the intensity of the peaks present in the sample before adsorption had decreased significantly. In addition, some minor shifts were also observed, which confirms that the present Amaranth dye adsorption onto the 6-ACA-CF surface was driven by the functional groups present on the 6-ACA-CF surface.

**Figure 3:**
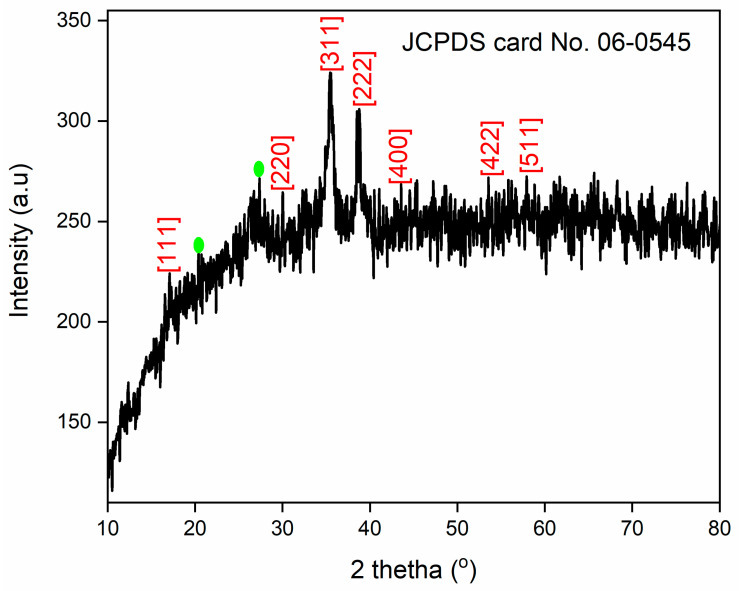
XRD of 6-ACA-CF NPs.

The X-ray diffraction (XRD) pattern of 6-ACA-CF (Figure 3) displayed characteristic peaks at 2θ values of 17.07, 30.09°, 35.47°, 38.82, 43.30°, 53.66°, 57.86°, and 63.43°, which belongs to the (111), (220), (311), (222), (400), (422), (511), and (440) planes of the cubic spinel structure of CF, as referenced by JCPDS card No. 06-0545 [39]. Additionally, for this study, the Crystal Impact Match program was used for the X-ray diffraction analysis [40]. This program showed that the sample contained Fe (46.7%), O (26.8%), and Cu (26.6%). The synthesized copper ferrite nanoparticles exhibited both cubic {Space group: F d −3 m; cell dimension: a = 8.3700 Å; I/Ic: 6.05} [Entry number: 96-901-2439] and tetragonal crystal system {Space group: I 41/a m d; cell dimension: a = 5.8000 Å and c = 8.7300 Å; I/Ic: 3.69} [Entry number: 96-901-1013], with the cubic phase being dominant (~80%) [41,42]. Some identified peaks were also observed due to the impurity. Additionally, the diffraction peaks marked with circles are associated with 6-amino caproic acid loaded on the CF [37].

The crystallite size of 6-ACA-CF NPs was calculated from the XRD data using Scherrer’s formula (Equation (3)).

(3) $$D=\frac{K\lambda }{\beta Cos\theta }$$

In this context, K = 0.9 is the shape factor, λ represent the wavelength of Cu Kα X-ray radiation (0.15418 Å), θ is the Bragg angle, and β indicate the full width at half maximum (FWHM) of the representative diffraction peak. The crystallite size, calculated using the most intense peak in the XRD pattern, was determined to be ~54.5 nm.

**Figure 4:**
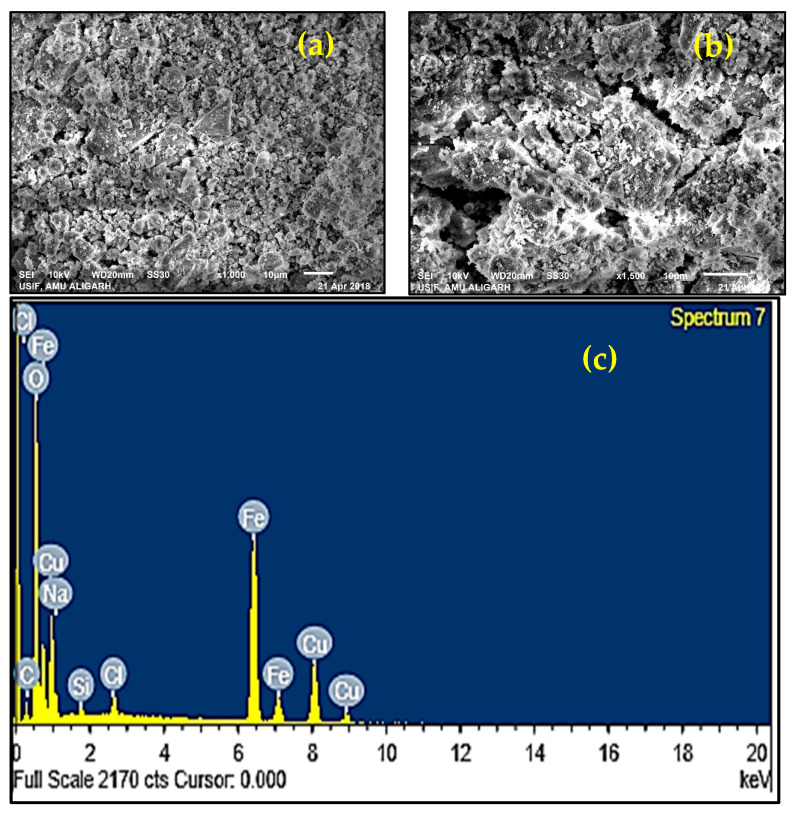
(**a**,**b**) SEM images of 6-ACA-CF NPs at different magnifications and (**c**) EDS spectra of 6-ACA-CF NPs.

The morphology of the 6-ACA-CF was confirmed by SEM analysis (Figure 4a,b). SEM image reveals roughness of 6-ACA-CF due to modification of CF NPs by 6-ACA that gives an amorphous structure. The agglomeration in particles can also be seen due to weak Vander Waal forces adhering between particles.

The elemental composition of 6-ACA-CF was confirmed with EDS analysis at the microscopic level. The results from the EDS pattern clearly confirmed the existence of elemental Cu, Fe, C, and O (Figure 4c). Na, Si, and Cl were present as impurities.

**Figure 5:**
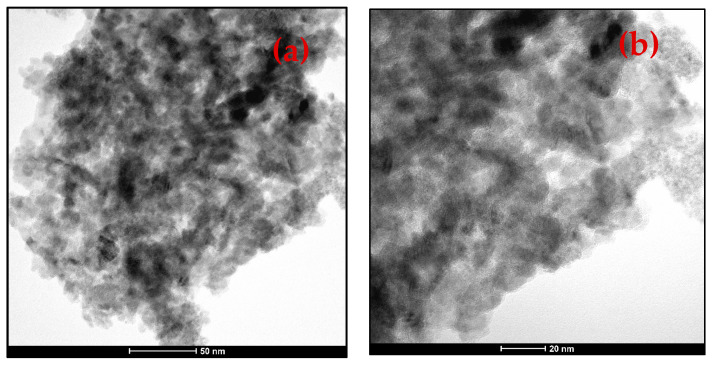
(**a**,**b**) TEM images of 6-ACA-CF at different magnifications.

The TEM image is used to show the internal morphology of the 6-ACA-CF sample, which revealed the distribution of nano-range particles of CF on 6-ACA (Figure 5a,b).

It’s worth noting that the sizes obtained by XRD and TEM differ significantly. XRD measures crystallite size using the Scherrer formula. TEM, on the other hand, displays particle sizes, which are formed by the aggregation of multiple crystallites, resulting in a larger size (than the XRD size). Furthermore, strain or defects can also affect the size in XRD. This is the main reason for the discrepancy between XRD and TEM sizes.

### 3.2. Adsorption Studies

The various sets of batch adsorption experiments were carried out to evaluate adsorption performance of 6-ACA-CF NPs for Amaranth dye. The results of these experiemnt are as follows.

#### 3.2.1. Effect of Adsorbent Dosage

**Figure 6:**
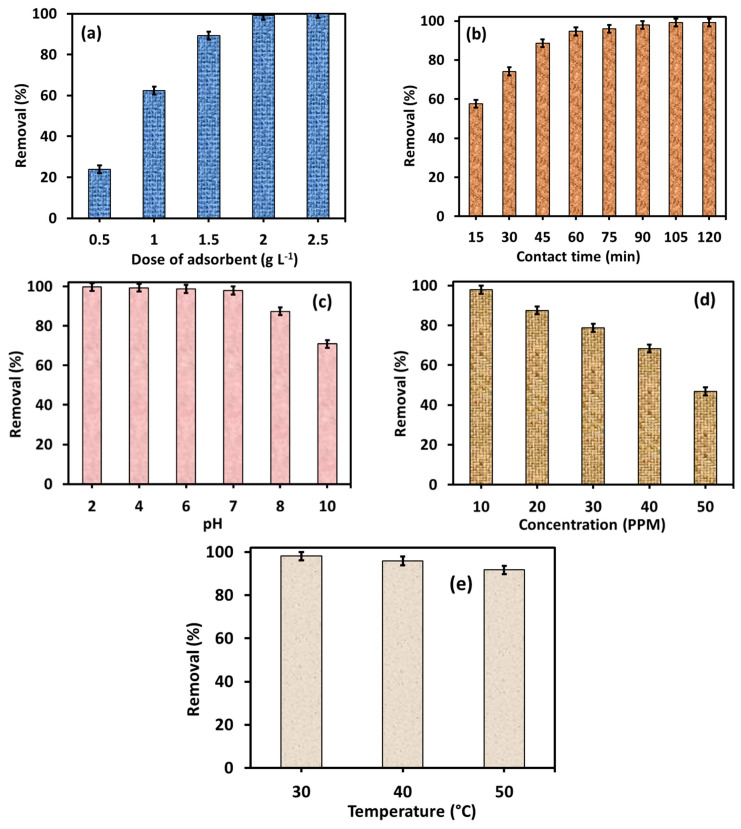
Optimization result of Amaranth dye adsorption under various variable changes: (**a**) 6-ACA-CF dose, (**b**) contact time, (**c**) solution pH, (**d**) concentration, and (**e**) temperature.

To evaluating the effect of dose of 6-ACA-CF NPs on the adsorption of Amaranth, a series of 50 mL Erlenmeyer flasks containing 10 mL of Amaranth solution of 10 mg L^−1^ concentration and different amounts of 6-ACA-CF NPs (1.0–2.5 g L^−1^) was stirred at 150 rpm for 120 min at pH 7 and temperature 30 °C. The results indicated in Figure 6a suggested that on increasing the dosage of 6-ACA-CF NPs sample from 1.0 to 2.5 g L^−1^ led to a raise in dye removal efficiency from ~20% to 100%. This improvement in removal (%) can be attributed to the greater availability of active adsorption sites as the amount of 6-ACA-CF sample increased. Since the dye concentration remained constant, the higher number of available sites enhanced the removal (%), respectively. The current results revealed that 2.0 g L^−1^ of 6-ACA-CF adsorbent was sufficient to remove ~99% of Amaranth dye from its 10 mg L^−1^ aqueous solution within 120 min at pH 7. For further studies, a 6-ACA-CF dose of 2.0 g L^−1^ was used.

#### 3.2.2. Effect of Contact Time

The duration of interaction between the adsorbate and the adsorbent plays an important role in determining the adsorption capacity and is essential for evaluating the kinetics of the adsorption process. This has also been investigated using 6-ACA-CF. Figure 6b revealed that with increased contact time, the sorption of Amaranth dye on 6-ACA-CF NPs surface increases gradually and attained ~95% removal at 60 min. In addition, ~98% of Amaranth dye adsorption onto the 6-ACA-CF surface was observed at 90 min. This behavior can be attributed to the initially available active sites on the surface of 6-ACA-CF NPs, which were initially unoccupied and readily accessible to Amaranth dye molecules. As a result, over 55% of the Amaranth dye is adsorbed within the first 15 min. As contact time increases, the probability of interactions between Amaranth dye molecules and the surface of the 6-ACA-CF also raises, resulting in a steady increase in adsorption performance of 6-ACA-CF for Amaranth dye removal. However, once all available adsorption sites become saturated, no further uptake occurs, and the system reaches equilibrium.

#### 3.2.3. pH Effect on Adsorption

It can be assumed that the interaction between the Amaranth dye and 6-ACA-CF may be significantly influenced by the surface charge of the adsorbent, which, in turn, is governed by the pH of the solution. The pH controls the adsorption behavior, likely due to surface deprotonation (–O^−^) under alkaline conditions and protonation (–OH_2_^+^) under acidic conditions [43,44,45]. For the current adsorption study, the pH of the dye solution plays a key role in regulating the adsorption performance of 6-ACA-CF for Amaranth dye removal, as illustrated in the corresponding Figure 6c. The adsorption performance of 6-ACA-CF for Amaranth dye removal was greater in acidic pH (2–6) in comparison to the alkaline environments (8–10) [44,45]. The graph shown continues declining in the adsorption performance of 6-ACA-CF for Amaranth dye removal from lower pHs to higher pHs. As earlier discussed, the changes in adsorption performance of any adsorbent are largely affected by the adsorbent’s pH_pzc_. The pH_pzc_ for 6-ACA-CF was found to be at ~7.9, which suggested slight negative surface of 6-ACA-CF NPs sample. Notably, the anionic Amaranth dye molecule is negatively charged; thus, at the pH < pH_pzc_, the surface of 6-ACA-CF NPs was probably positively charged and therefore attracted the anionic Amaranth dye molecule more effectively. When pH > pH_pzc_, the surface of the 6-ACA-CF NPs may have been negatively charged, which led to deprotonation, thereby repelling the anionic Amaranth dye [44]. Therefore, the higher pH does not favor the adsorption of Amaranth onto the 6-ACA-CF NPs surface, while lower pHs favor the current adsorption.

#### 3.2.4. Effect of Initial Concentration

The adsorption of Amaranth dye onto 2.0 g L^−1^ of 6-ACA-CF dose was further investigated by varying the initial dye concentration from 10 to 50 mg L^−1^. It was observed that the amount of dye adsorbed per gram of NPs (mg g^−1^) increased with raising initial dye concentration from 10 to 50 mg L^−1^ (Figure 6d). This result can be attributed to the greater availability of dye molecules in the solution, which enhances the likelihood of collisions and interactions with the available active sites on the 2.0 g L^−1^ of 6-ACA-CF sample surface. The increase in concentration also contributes to a stronger concentration gradient, which acts as a driving force for adsorption. As the adsorption sites become fully occupied, the system reaches equilibrium after a certain concentration. This study further revealed that a dose of 2.0 g L^−1^ of 6-ACA-CF sample was sufficient to remove over ~80% of Amaranth dye at an initial concentration of 30 mg L^−1^ (at pH 7, contact time 90 min, and temperature 30 °C). However, when the initial concentration was further increased to 50 mg L^−1^, the removal (%) was significantly dropped to ~47%. This result is likely due to the limited number of active adsorption sites being insufficient to accommodate the larger number of dye molecules with increasing concentration.

#### 3.2.5. Thermodynamics

The effects of reaction temperature were demonstrated at three variable temperatures, 30, 40 and 50 °C, with 10 mg L^−1^ of Amaranth initial concentration, and 2.0 g L^−1^ dosage of 6-ACA-CF NPs. The effect of reaction temperature on the adsorption performance of 6-ACA-CF NPs for Amaranth dye removal is shown in Figure 6e. It can be revealed that with the increase in temperature from 30 to 50 °C, the adsorption performance of 6-ACA-CF NPs for Amaranth dye removal is decreased. This effect might be due to the weakening of interaction between Amaranth molecules and 6-ACA-CF NPs surface; therefore, the process of sorption of Amaranth onto the 6-ACA-CF surface was reported to be exothermic.

To further get into insight mechanism, the thermodynamics parameters such as change in Gibbs free energy (ΔG), entropy change (ΔS), and enthalpy change (ΔH), have also been calculated utilizing temperature-dependant Amaranth dye adsorption data. These parameters demonstrate the heat change during reaction, feasibility, and spontaneity of the reaction.

The ΔG of the adsorption process is related to constant, Kc (Equation (4)) [25].

(4) ∆G=RTlnKc

ΔG value was calculated from Equation (5). The ΔH and ΔS values were evaluated utilizing the Van’t Hoff equation [25]:

(5) ∆G=∆H−T∆S

**Figure 7:**
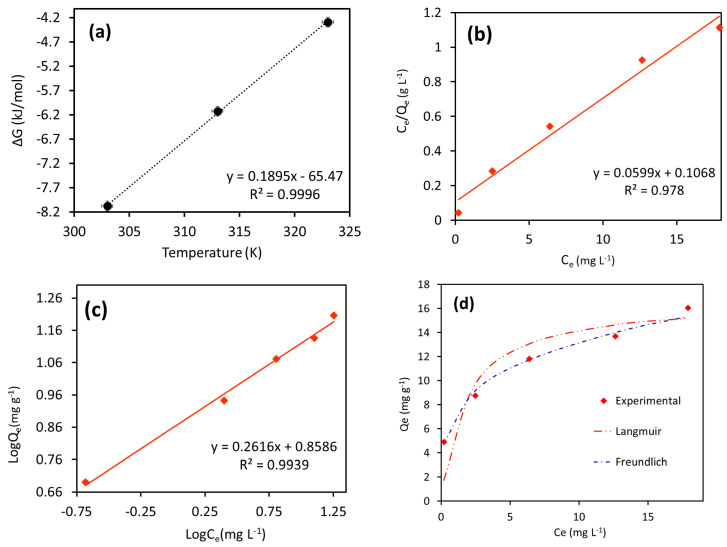
Plot of (**a**) thermodynamics, (**b**) Langmuir isotherm, (**c**) Freundlich isotherm, and (**d**) isotherm simulation for Amaranth dye adsorption onto the 6-ACA-CF (experimental conditions: 6-ACA-CF dose = 2.0 g/L; Amaranth concentration = 10.0 mg/L; Temperature = 30 °C; Contact time = 90 min; pH = 7.0; Agitation speed = 150 rpm).

The intercept and slope of the plot between ∆G and T (Equation (5)) gave the values of ∆H and ∆S, respectively. The ∆G values for current adsorption were observed −8.08, −6.12, and −4.30 kJ mol^−1^ at 30, 40, and 50 °C, respectively (Figure 7a; Table 1a), demonstrating the thermodynamically viable and spontaneous adsorption process. Further, the ∆G values ranged from 0 to −20 kJ mol^−1,^ confirming the predominant physical sorption of Amaranth dye onto the 6-ACA-CF surface. The value of ∆H was calculated to be −65.47 kJ mol^−1^, confirming the exothermic process with a probability of a favorable adsorption. The entropy value of −0.189 J mol^−1^ K^−1^ was corresponded to a lowering in the degree of freedom of the system, indicating adsorption of Amaranth dye onto the 6-ACA-CF surface.

#### 3.2.6. Isotherms

The temperature-dependant experimental data were analyzed for their suitability with adsorption isotherm models, specifically the Langmuir and Freundlich isotherms.

##### Langmuir Isotherm

Langmuir adsorption is based on the isothermal hypothesis which includes adsorption that occurs uniformly on a homogeneous and energetically equivalent adsorbent surface and forms a monolayer.

The linearized form of the Langmuir isotherm, as described in reference [25], is given by (Equation (6)):

(6) $$\frac{C_{e}}{Q_{e}}=\frac{C_{e}}{Q_{o}}+\frac{1}{Q_{o}b}$$

Here, *Q_o_* (mg/g) denotes the adsorbent maximum adsorption capacity that is the amount of adsorbate essential to form a complete monolayer on the adsorbent surface. *Q_e_* (mg/g) denotes the adsorption capacity at equilibrium, while b (L/mg) is the Langmuir constant related to the adsorption energy or the affinity between the adsorbent and the adsorbate. The values of *Q_o_* and b for Amaranth dye removal were calculated from the linear plot of *C_e_/Q_e_* versus *C_e_* using slope and intercept of the plot at 30 °C (as presented in Table 1b and Figure 7b).

At the temperature 30 °C, the *Q_o_* value for Amaranth dye was 16.70 mg g^−1^. Additionally, the *b* value was found to be 0.56 L mg^−1^, at 30 °C, suggesting a stronger binding affinity of Amaranth dye to the 6-ACA-CF NPs at this temperature. The practicality of the adsorption process was evaluated using the dimensionless separation factor (R_L_), which was determined using the following Equation (7):

(7) $$R_{L}=\frac{1}{1+bC_{e}}$$

The R_L_ value, known as the separation factor, represent the feasibility of the adsorption method. An R_L_ value greater than 1 suggests that adsorption is unfavorable, while an R_L_ of 0 implies an irreversible process. When R_L_ falls between 0 and 1, the adsorption is considered energetically favorable. In the case of current Amaranth adsorption, R_L_ value was found to be within this favorable range (0 < R_L_ < 1) at the tested temperature, confirming the viability of the adsorption process under the experimental conditions [24,25]. The coefficient of determination (R^2^) for Amaranth dye was 0.978.

##### Freundlich Isotherm

The Freundlich adsorption isotherm characterizes adsorption on both homogeneous and heterogeneous surfaces of an adsorbent. It considers the possibility of both monolayer adsorption (chemisorption) and multilayer adsorption (physisorption). The linearized equation for the Freundlich isotherm is given by Equation (8) [25]:

(8) $$logQ_{e}=logK_{F}+\frac{1}{n}logC_{e}$$

The Freundlich isotherm constants *K_F_* [(mg/g)(L/mg)^1/n^] and n denote the adsorption capacity and intensity, respectively. Specifically, *K_F_* indicates the capacity for multilayer adsorption at a unit concentration of adsorbate and serves as a relative indicator of the adsorbent’s adsorption ability. The constant *n* reflects the intensity of adsorption and is influenced by adsorbent surface heterogeneity. Both *K_F_* and n (as presented in Table 1b) were derived from the slope and intercept of the linear plot of log*Q_e_* versus log*C_e_* (Figure 7c).

For adsorption to be considered favorable, *n* should typically fall within the range of 1 to 10. Lower values of *n* (approaching zero) suggest increased heterogeneity of the surface. A higher n value (and thus a lower 1/n) indicates stronger interactions between adsorbate and adsorbent. When 1/n is less than 1, it corresponds to typical L-type isotherm behavior, while values greater than 1 suggest cooperative adsorption behavior.

From the Freundlich plots (Figure 7b), *K_F_* value was found to be 7.22 [(mg g^−1^)(L mg)^−1/n^], and *n* value was 3.82 at 30 °C, as detailed in Table 1b. The observed value of n suggested the physical adsorption of Amaranth dye onto the heterogeneous surface of 6-ACA-CF. The Freundlich model showed R^2^ value at 0.993.

Based on the linearity of the plots, it can be said that the Freundlich isotherm showed better results with the existing adsorption data than the Langmuir isotherm. Furthermore, when the experimental adsorption data was compared with the theoretical adsorption data of the Langmuir and Freundlich isotherm in simulation plot (Figure 7d), it was found that the values in the Freundlich isotherm showed greater similarity to the experimental values, which led to a lower error function observed for the simulation plot of the Freundlich isotherm. This meaning that the Freundlich isotherm plot was best fit plot for the present adsorption data. Therefore, it can be said that the current adsorption of Amaranth dye onto the surface of 6-ACA-CF was multi-layered, physical, and heterogeneous.

#### 3.2.7. Kinetics

To gain a clearer understanding of the adsorption kinetics in this study, the experimental data for the removal of Amaranth dye from aqueous solutions were analyzed using two well-established kinetic models: the pseudo-first-order (PFO) and pseudo-second-order (PSO) models [25,46].

The PFO kinetic model is commonly applied to describe adsorption processes in liquid–solid systems. It presumes that the adsorption rate is directly dependent on the abundance of vacant binding sites on the adsorbent surface. The model is typically expressed using a non-linear mathematical equation (Equation (9)).

(9) $$Q_{t}=Q_{e}\;(1-e^{K_{1}t})$$

Here, *Q_t_* represents the adsorption efficiency at time t and *Q_e_* (mg g^−1^) at equilibrium, K_1_ (minutes^−1^) is PFO rate constant for adsorption.

The PSO kinetic model is based on the assumption that the adsorption rate depends on the number of available active sites on the adsorbent surface as well as the concentration of adsorbate molecules in the solution. It implies that the adsorption rate is proportional to the square of the difference between the equilibrium number of adsorption sites and the number of sites already occupied. This model also implies that the rate-limiting step leads the chemical bonds formation between the adsorbate and adsorbent at specific active sites.

This can be expressed by the following non-linear form of PSO kinetic model represented as (Equation (10)):

(10) $$Q_{t}=\frac{K_{2}Q_{e}^{2}}{1+\;K_{2}Q_{e}t}$$

where K_2_ represents overall rate constant for adsorption process. The kinetic parameters for Amaranth dye adsorption were obtained from the slope of the linear plot of t/Q_t_ versus t at 30 °C.

Another kinetic model named Elovich, was also utilized to explain the kinetics of present adsorption process. The Elovich model is used to understand chemical adsorption and to describe adsorption and desorption rate. This model can be demonstrated by the non-linear mathematical equation (Equation (11)).

(11) $$Q_{t}=1/\beta ln(\alpha \beta t+1)$$

where α and β are the rate of adsorption and desorption, respectively.

As previously established, the current adsorption process is multi-step in nature, suggesting that the transfer of Amaranth dye molecules from the solution to the solid surface may involves intraparticle diffusion (IPD) and film diffusion (FD) mechanisms. Therefore, the present adsorption mechanism validated by the experimental kinetic data were demonstrated utilizing Weber–Morris (WM) model, which can be described by Equation (12).

(12) $$Q_{t}=K_{ipd}t^{0.5}+C$$

Here, K_ipd_ represents the intraparticle diffusion rate constant and is equal to the slope and C is a thickness of boundary layer represent by intercept. Ideally, this plot should pass through the origin, indicating that ID is the only rate-controlling step, otherwise both the mechanism applies, and then C value found.

**Figure 8:**
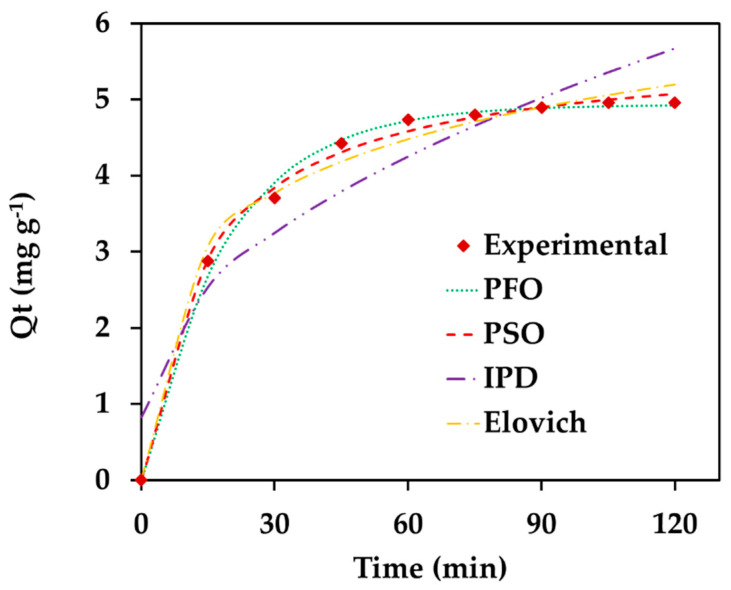
Plot of adsorption kinetics for Amaranth dye adsorption onto the 6-ACA-CF (Experimental conditions: 6-ACA-CF dose = 2.0 g/L; Amaranth concentration = 10.0 mg/L; Temperature = 30 °C; pH =7.0; Agitation speed = 150 rpm).

To evaluate the goodness of fit between the kinetic model and the experimental adsorption result, both the error functions (Sum of Square Root, SSR and Chi-square, χ2) and model variable were calculated (Figure 8), with the results summarized in the accompanying table (Table 2).

As listed in the table (Table 2), the lowest error functions were obtained for the PSO model with the best fit agreement to experimental data, suggesting it provides a more accurate representation of the adsorption mechanism under the tested conditions. Elovich model suggested the higher adsorption rate in comparison e to the desorption rate (α > β) along with the good fitting to adsorption data, revealed chemical adsorption.

The WM plot gives the, *K_ipd_*, and C value that was found to be 0.4419 mg g^−1^ min^0.5^ and 0.8268 (intercept). The WM model results indicated that the rate of current adsorption process may have affected through both IPD as well as FD, suggesting that the present process was multi-step.

The collective results of thermodynamics, isotherms, and kinetics revealed a multilayer, phyco-chemical, adsorption of Amaranth dye onto the surface of 6-ACA-CF.

#### 3.2.8. Regeneration and Reutilization

**Figure 9:**
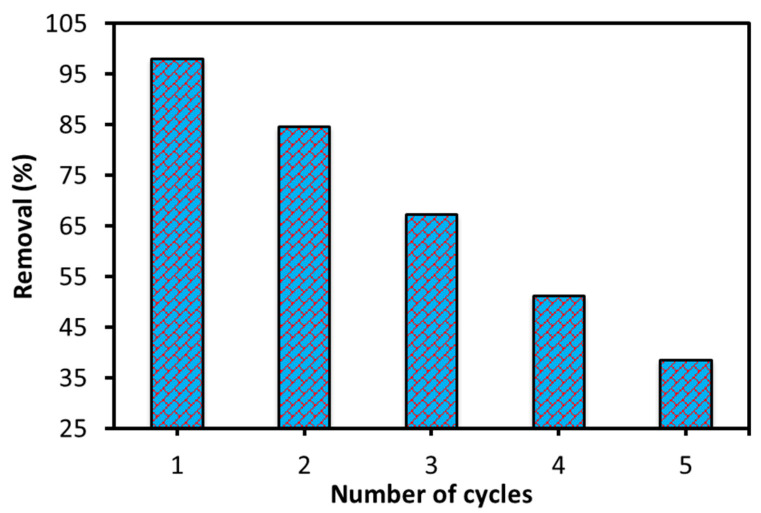
Plot of adsorption–desorption cycles for Amaranth dye adsorption onto the 6-ACA-CF.

The current adsorbent also tested for the stability and reusability via regeneration and adsorption–desorption cycles. In brief, the dye-loaded adsorbent was first collected and then immersed in a 0.1 M solution of NaOH, and magnetically stirred for 4 h. The adsorbent was then separated from the solution and washed until the pH of the solution became neutral. It was then dried and used for the next adsorption cycle. The regeneration and re-adsorption process was repeated several times (up to five cycles), and the results were noted. The results of this optimization cycle are shown in Figure 9. This confirms that the current adsorbent performed well for two cycles. After that, its adsorption performance gradually decreased, and efficiency decreased by approximately 60% after the five cycles.

It is important to note that some adsorbent is lost during regeneration after each cycle, reducing the adsorbent’s available effective mass. Furthermore, functional groups on the adsorbent’s surface may be damaged, or some dye molecules may become strongly bound to the surface through chemical adsorption, leading to overall blockage. It is also possible that repeated regeneration may have caused NPs to aggregate, reducing surface area and making the dye molecules less accessible to surface. Sometimes, by-products or impurities from previous cycles also interfere with adsorption. All these factors reduce the adsorption efficiency of 6-ACA-CF after the first or second cycle. Therefore, it can be concluded that this adsorbent remained efficient for the first two cycles.

#### 3.2.9. Interfering Molecules/Ions Effect

**Figure 10:**
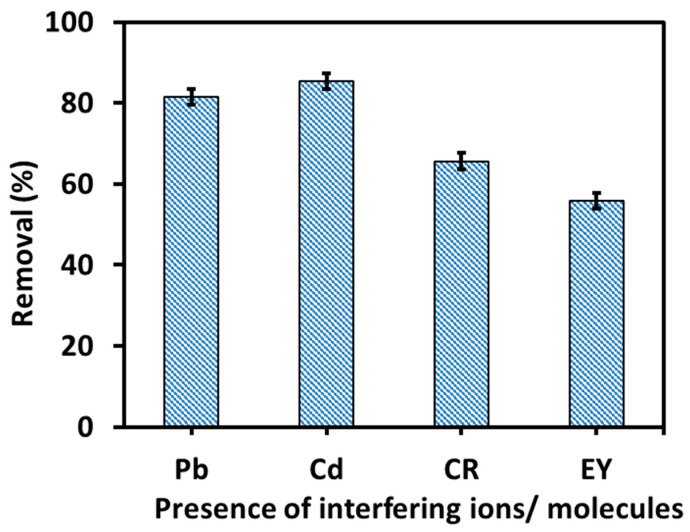
Competitive ion/molecules effect on Amaranth dye removal.

Water contains several organic/inorganic substances, which affect the efficiency of dye removal during the adsorption process. Therefore, the interfering molecules/ions effect was also investigated in this study. For this purpose, the adsorption experiment was carried out under the same experimental conditions with metal ions (Pb^2+^ and Cd^2+^) and dye molecules (Congo red (CR) and Eosin yellow (EY)) (Figure 10). The results of the competitive ions/molecules effect showed that the presence of anionic dyes such as (CR and EY) significantly affected the adsorption of the Amaranth dye, mainly due to competition between the anions. This was primarily because some positively charged adsorption sites of 6-ACA-CF (at pH = 7 < pH_pzc_) were occupied by the other anionic dyes, which reduced the removal efficiency of the dye. The EY dye showed a more competitive effect compared to CR, likely because EY (molecular weight = 691.88) has a smaller size than CR (molecular weight = 696.66), which could lead to higher diffusion of EY on the 6-ACA-CF surface. The presence of cations/MB also affected the removal efficiency; however, the effect was not significant. The effect of cations was as follows: Pb^2+^ > Cd^2+^. Pb^2+^ has higher electronegativity and a smaller hydrated ionic radius compared to Cd^2+^, resulting in higher affinity and diffusion rates, which is responsible for greater interference of Pb^2+^ with Amaranth adsorption onto the 6-ACA-CF surface.

#### 3.2.10. Comparative Studies

Based on various studies and experiments conducted to determine the effectiveness of different adsorbents in removing amaranth dye from aqueous solutions, it can be concluded that the present adsorbent revealed the significant performance adsorption for the Amaranth dye. The comparative studies are given in the form of Table 3.

## 4. Conclusions

In this paper, 6-ACA-CF (CuFe_2_O_4_ functionalized with 6-aminocaproic acid) nanoparticles were successfully synthesized by the coprecipitation method. The synthesis of the nanocomposite and its adsorption of Amaranth dye were verified using various characterization method, for example, XRD, SEM-EDS, FTIR, and TEM. Batch experiments were conducted to investigate the removal of Amaranth dye using 6-ACA-CF nanoparticles, focusing on the influence of various operational parameters. The adsorption behavior and underlying mechanisms were analyzed through thermodynamics and isotherm models. The optimal pH range for effective dye removal was found to be between 2 and 6. The adsorption equilibrium data fit well with the Freundlich isotherm, revealing a multilayer, and heterogeneous adsorption. The kinetic model suggested the pseudo second-order adsorption process governed through the multi-step process. The maximum adsorption capacity of 6-ACA-CF for Amaranth dye was reported to be ~17 mg g^−1^. The stability and reusability results suggested the best performance of current adsorbent for first two adsorption–desorption cycles.

## Figures and Tables

**Table 1:** (**a**) Results of thermodynamic parameters. (**b**) Results of isotherm parameters.

(**a**)				
Parameters	Temperatures (K)			
	303	313		323
Q_e_ (mg g^−1^)	~4.90	~4.80		~4.60
∆G (kJ mol^−1^)	−8.08	−6.12		−4.30
∆H (kJ mol^−1^)	−65.47			
∆S (kJ mol^−1^·K^−1^)	−0.189			
(**b**)				
Isotherms	Parameters		Values	
Langmuir	Q_o_		16.70	
	b		0.56	
	R_L_		0.151	
	R^2^		0.978	
	ARE		19.70	
Freundlich	kF		7.22	
	n		3.82	
	R^2^		0.993	
	ARE		2.88	

**Table 2:** Results of Kinetic parameters.

Kinetics	Parameters	Values
PFO	K_1_	0.0524
	Q_e_	4.9266
	SSR	0.0851
	χ^2^	0.0024
PSO	K_2_	0.0123
	Q_e_	5.6757
	SSR	0.0700
	χ^2^	0.0019
WM	K_id_	0.4419
	C	0.8268
	SSR	2.3490
	χ^2^	0.0664
Elovich	α	1.2430
	β	0.9559
	SSR	0.2370
	χ^2^	0.0067

**Table 3:** Comparative analysis of adsorbents.

S. No.	Adsorbent Name	Q_max_ (mg g^−1^)	Reference
1.	Guava leaves	1.028	[47]
2.	Potato peels	1.709	
3.	Iron oxide NPs coated with cetyltrimethylammonium bromide	1.05	[43]
4.	Eggshell powder	2.032	[44]
5.	Bottom ash	7.80	[45]
**6.**	**6-ACA-CF**	**16.70**	**This study**

## Data Availability

The original contributions presented in this study are included in the article. Further inquiries can be directed to the corresponding authors.
